# Post-traumatic stress disorder symptoms in children after PICU discharge: exploring contributing factors and the need for targeted interventions

**DOI:** 10.1186/s12888-026-08079-w

**Published:** 2026-04-24

**Authors:** Mao-Ting Tang, Xian-Liang Liu, Hai-Yang Zhang, Ping Lei Chui, Mei Chan Chong, Lin Mo

**Affiliations:** 1 Department of Outpatient Children’s Hospital of Chongqing Medical University, National Clinical Research Center for Children and Adolescents’ Health and Diseases, Ministry of Education Key Laboratory of Child Development and Disorders, International Science and Technology Cooperation Base of Child Development and Critical Disorders, Chongqing Key Laboratory of Child Neurodevelopment and Cognitive Disorders, Chongqing, China; 2 School of Nursing and Health Studies, Hong Kong Metropolitan University, Homantin, Kowloon, Hong Kong SAR China; 3 West China Second University Hospital, Sichuan University, Chengdu, Sichuan China; 4 Department of Nursing Science, Faculty of Medicine, Universiti Malaya, Kuala Lumpur, Wilayah Persekutuan, Kuala Lumpur, 50603 Malaysia

**Keywords:** Post-traumatic stress disorder, Pediatric intensive care unit, Qualitative study

## Abstract

**Background:**

The rising number of pediatric intensive care unit (PICU) admissions has brought post-traumatic stress disorder (PTSD) into sharp focus as a pressing mental health concern among pediatric patients. This study aimed to explore the PTSD-related experiences of children following PICU hospitalization.

**Methods:**

This descriptive qualitative study investigated PTSD-related experiences in children aged 8–18 years discharged from PICUs in Chengdu, China. Using stratified purposive sampling, we conducted in-depth interviews with participants one-month post-discharge. Data were analyzed thematically through coding, categorization, and theme extraction. To ensure rigor, we employed triangulation, member checking, and expert review.

**Results:**

Interviews with 11 children (six boys and five girls) revealed significant PTSD symptoms after PICU discharge. Key contributors to PTSD include physical health and sleep disturbances, psychological distress, environmental stressors, invasive medical interventions, and a lack of social interaction.

**Conclusions:**

Post-PICU PTSD symptoms in children are shaped by a combination of physical, psychological, and social factors. Tailored interventions are urgently needed to mitigate these risks, emphasizing holistic care to support recovery and mental well-being.

**Supplementary Information:**

The online version contains supplementary material available at 10.1186/s12888-026-08079-w.

## Background

The pediatric intensive care unit (PICU) is a high-stakes environment where life-saving interventions, such as emergency resuscitation, mechanical ventilation, and invasive procedures, are performed under immense pressure [1, 2]. While these treatments aim to preserve life, they can also cause significant physical and psychological trauma in children. The sudden onset of illness, combined with fear and exposure to complex medical procedures, leaves young patients particularly vulnerable to post-traumatic stress disorder (PTSD) after discharge. Symptoms such as intrusive memories, emotional instability, hypervigilance, and recurring nightmares may emerge within the first month post-discharge, often escalating into long-term psychological issues such as anxiety, depression, sleep disorders, and impaired social or academic functioning if left unaddressed [3–6].

In pediatric PICU survivors, these symptoms disrupt daily activities, strain family relationships, hinder academic performance, and challenge social adaptation [7, 8]. Despite growing attention to PTSD in PICU survivors, children often struggle to articulate their emotions or connect physiological responses to trauma, making early identification and intervention difficult. Understanding specific symptoms and their impact during the critical first month post-discharge is essential for addressing mental health needs.

While previous studies have quantified the prevalence and predictors of PTSD in PICU survivors [7, 9, 10], they often relied on standardized questionnaires that fail to capture the depth of children’s subjective experiences. Developmental limitations in cognition and expression further complicate efforts to explore children’s emotional responses, coping mechanisms, and interactions with their environment using quantitative methods. Standardized measures may overlook context-dependent nuances of trauma, limiting their ability to fully account for the unique and individualized nature of children’s experiences.

In contrast, qualitative inquiry enables a deeper exploration of these complexities. By using methods such as in-depth interviews and observations, qualitative research provides a nuanced understanding of how children interpret and navigate their trauma. This approach uncovers psychological processes and emotional responses that remain hidden in numerical data, offering unique insights into individual coping and meaning-making processes. Through this lens, the study sheds light on aspects of trauma that quantitative approaches may fail to address, enriching our understanding of PTSD in pediatric patients.

By bridging the gap between statistical trends and individual experiences, qualitative studies have advanced our understanding of PTSD in pediatric patients. Qualitative research has laid the groundwork for early identification of high-risk children, refinement of assessment tools, and development of personalized interventions. This approach not only enhances mental health outcomes but also informs the design of holistic care strategies that address the unique vulnerabilities of children recovering from the PICU experience. Therefore, this study aims to investigate the factors during PICU stays that contribute to PTSD symptoms development in children one month post-discharge.

## Methods

### Ethical considerations

This study was approved by the Medical Ethics Committee (approval number: 2023 − 254) and conducted in accordance with the 1975 Declaration of Helsinki. Written informed consent was obtained from parents or legal guardians, and assent was secured from children based on their age and understanding. Participant privacy and data confidentiality were strictly maintained, with all data anonymized and used solely for research purposes.

### Design

This descriptive qualitative study aimed to explore the experiences of children with PTSD symptoms one month after discharge from the PICU and to identify factors contributing to its development. Qualitative research is particularly effective for examining underexplored phenomena or areas lacking systematic study [11]. Given the limited research on PTSD in PICU patients, this approach enabled a comprehensive understanding of their lived experiences and how this influences the development of PTSD. To ensure transparency and rigor throughout the study, including interview design, data collection, and analysis, the Consolidated Criteria for Reporting Qualitative Research (COREQ) guidelines were meticulously followed [12]. This methodological framework provides a robust structure for capturing nuanced insights into the unique experiences of this vulnerable population. Since no interventions were applied, this study is not a clinical trial. Clinical trial number: not applicable.

### Setting and participants

This study was conducted in Chengdu, China. To ensure geographic representation, the 12 local PICU hospitals were divided into four regions: north (three hospitals), south (three hospitals), west (four hospitals), and east (two hospitals). One hospital from each region was randomly selected as the study site. This regional stratification ensures that variability in hospital environment and resource availability is captured in the sample.

Participants were eligible if they met the following criteria: aged 8–18 years, awake, able to communicate verbally, hospitalized in the PICU for at least 48 h, and screened as high-risk for PTSD (Children’s Revised Impact of Event Scale, CRIES ≥ 31) one month after discharge. Participation also required parental or guardian consent, along with signed informed consent from the child.

Exclusion criteria included death within one-month post-discharge, inability to contact the family, rehospitalization in the PICU within one month, or a history of pre-existing mental health conditions.

The research team employed purposive sampling to recruit participants, inviting 25 children one month after PICU discharge to participate in interviews. However, 14 invitees could not participate due to parental refusal (*n* = 6), inability to be contacted (*n* = 4), or scheduling conflicts (*n* = 4). Ultimately, 11 children representing each age group accepted the invitation and completed the interviews, ensuring diverse age representation. The interviews were conducted between September 2023 and January 2024, with data saturation achieved by the final interviews [13]. Based on existing qualitative standards and the attainment of thematic redundancy by the 11th interview, the sample size was deemed adequate. To maintain objectivity during data collection, the research team had no prior relationships with the participants.

### Data collection

The interviews were conducted using a semi-structured interview guide (see Additional File 1, Table A) to investigate the subjective experiences of children with PTSD symptoms one month after discharge and to identify factors contributing to PTSD symptoms during their PICU hospitalization. The interview content included background information (such as age, length of hospitalization, medical history, and family background) and focused on the children’s psychological experiences in the PICU, emphasizing their perceptions of the hospital environment, physical and emotional responses, and sleep conditions. The interview questions were developed based on a thorough review of existing literature [14, 15] and the researcher’s expertise in PICU nursing. To ensure scientific rigor and relevance, two doctoral nursing supervisors reviewed and refined the questions prior to the interviews. One month after discharge, the researchers contacted the children’s parents via phone or text to confirm the interview time and location.

The interviews were conducted in a quiet, private room within the hospital to ensure an undisturbed environment. Parents observed from outside through a transparent glass window to minimize interference while maintaining their presence. The interviewer, a highly qualified nursing professional with a PhD, six years of PICU experience, and specialized training in qualitative research, brought extensive expertise in pediatric critical care and psychological needs. Her experience in conducting qualitative interviews and data analysis ensured a structured and professional approach.

With participants’ consent, all interviews were audio-recorded and kept confidential. Each interview lasted 20–30 min, during which the interviewer actively listened, refrained from interrupting, and avoided expressing personal opinions. Nonverbal behaviors, including facial expressions, body language, and moments of silence, were carefully observed to gain deeper insights into children’s emotional states. The interview process was flexible, with questions naturally adapted based on participants’ responses to encourage smooth and meaningful discussions. To enhance data accuracy and credibility, transcripts were returned to participants for review, allowing them to provide feedback and make corrections. In cases where participants showed signs of emotional distress, the researchers offered appropriate psychological support to ensure their well-being and prevent secondary psychological harm.

### Data analysis

The data were analyzed using Creswell’s thematic analysis method [16], following a series of systematic steps to ensure rigor and reliability. Initially, the primary researcher (TMT) conducted face-to-face interviews and transcribed the discussions verbatim in Chinese. To maintain accuracy and consistency, CMC translated the transcripts into English and verified their quality. To gain a deep understanding of the data, the primary researcher (TMT) thoroughly and repeatedly read the transcripts, reflecting on the content to identify emerging themes. The second researcher (CPL) independently reviewed the data, providing critical feedback and additional insights. This collaborative approach enhanced the reliability of the analysis and ensured a comprehensive interpretation of the findings.

In the initial coding phase, the primary researcher (TMT) systematically identified and categorized meaningful units within the text. The second researcher (CPL) conducted cross-validation to ensure consistency and accuracy in coding. This process was supported by NVivo 12 software, which facilitated the organization, management, and systematic analysis of the data. Themes were developed by the primary researcher (TMT) through refinement and synthesis of the preliminary coded data. Common patterns were identified using tools such as frequency analysis and keyword extraction. The second researcher (CPL) evaluated the coherence and relevance of the themes and provided constructive feedback to enhance clarity and precision. To further ensure credibility, the primary researcher (TMT) reviewed the integrity and logical consistency of the data, while the second researcher (CPL) performed cross-checks. Additionally, the translator (CMC) conducted a final validation to confirm the accuracy and clarity of the English translation. Through this meticulous process, the analysis ensured the reliability and validity of the findings [17, 18]. To enhance analytical rigor and credibility, investigator triangulation was employed, whereby all researchers independently analyzed the data and then collaboratively discussed their interpretations to ensure the reliability and consistency of the findings. Table 1 provides illustrative examples of the sequential steps involved in the data analysis.

**Table 1:** Examples of the data analysis

Repeatedly read the textual materials.	Code	Synthesize themes
My throat hurts so much! It feels like there’s a sharp knife stabbing inside.	Severe throat pain	Physical health issues
This really worried me because it’s the first time I’ve ever needed external help to breathe.	Fear of medical intervention	Psychological distress
Every night, I hear the piercing alarms of the monitors, the mechanical sounds of the infusion pumps, and the rumbling noises of equipment being pushed by medical staff.	Environmental noise	Environmental stressors
Although I feel very tired, it is difficult for me to fall asleep in this constantly disturbed environment.	Sleep difficulties	Sleep problems
When treatment begins, the nurses adjust my position, change my medication, or insert tubes, often requiring me to stay awake.	Treatment procedures	Medical intervention
Although my family visits me occasionally, their time is always so short, and the rest of the time, I’m left alone in the hospital room.	Limited family interaction	Lack of social interaction

## Results

### Participant characteristics

Eleven children (six boys and five girls), aged 8 to 18 years, participated in the interviews. Each child represented a different age group and exhibited severe PTSD symptoms following discharge from the PICU (CRIES score ≥ 31). Most of the children were in elementary or junior high school. The mothers’ educational backgrounds varied, ranging from junior high school, high school, or vocational school to college-level education or higher. Regarding family structure, the participants came from diverse settings, including single-parent, incomplete, and extended families. The duration of PICU stay ranged from 2 to 21 days. Table 2 provides detailed demographic and clinical information on the participants.

**Table 2:** Demographic characteristics of the participants (*N* = 11)

Serial number	Age	Sex	Household per capita monthly income (yuan)	Ethnicity	Children’s education level	Mother’s educational level	Family types	Length of stay in the PICU (day)
A1	8	Boy	10,001–20,000	Han	Primary school	Junior high school	Single parent	2–7
A2	9	Girl	5,001–10,000	Han	Primary school	Primary school	Incomplete	2–7
A3	10	Boy	5,001–10,000	Han	Primary school	Junior high school	Single parent	15–21
A4	11	Boy	10,001–20,000	Others	Primary school	Junior high school	Extended	8–14
A5	12	Girl	1,000–5,000	Han	Primary school	High school or vocational school	Incomplete	2–7
A6	13	Girl	5,001–10,000	Tibetan	Primary school	Primary school	Nuclear	15–21
A7	14	Boy	1,000–5,000	Han	Junior high school	Junior high school	Single parent	8–14
A8	15	Girl	5,001–10,000	Han	Junior high school	University and above	Incomplete	15–21
A9	16	Girl	10,001–20,000	Hui	Junior high school	High school or vocational school	Incomplete	15–21
A10	17	Boy	10,001–20,000	Han	High school or vocational school	University and above	Single parent	2–7
A11	18	Boy	5,001–10,000	Hui	High school or vocational school	High school or vocational school	Extended	15–21

Note: CRIES, Children’s revised impact of event scale; PICU, pediatric intensive care unit; PTSD, post-traumatic stress disorder

### Themes

Using Creswell’s method for thematic analysis [16], supported by rigorous cross-validation of coding and the use of NVivo software for data organization, one overarching theme emerged from the interviews: factors affecting PTSD symptoms in children in the PICU. This main theme was further categorized into six subthemes: physical health issues, psychological distress, sleep problems, environmental stressors, medical interventions, and lack of social interaction. The subthemes are presented in order of their frequency of mention by participants, reflecting the relative prominence of each factor in contributing to PTSD symptoms in children in the PICU.

### Physical health issues

Children in the PICU often face complex physical health challenges, including acute and severe chronic illnesses [19]. Common issues include severe pain (e.g., postoperative pain or pain caused by the underlying disease), physical weakness (resulting from prolonged bed rest, infections, or malnutrition), and disease progression (e.g., multi-organ dysfunction or acute exacerbations) [19]. These physical difficulties not only impede the child’s physical recovery but also exacerbate their psychological burden, creating a compounding effect that may significantly affect the overall rehabilitation process [20].

*I feel like a lump of dough*,* soft and limp*,* with no strength. Moving my arm is like lifting a heavy stone; I can’t lift it at all. (A7*,* Boy*,* 14 years)*

*My throat hurts so much! It feels like there’s a sharp knife stabbing inside. Every swallow is extremely uncomfortable*,* as if there’s fire burning in my throat*,* my voice has become hoarse. (A2*,* Girl*,* 9 years)*

*My wound hurts so much*,* especially when I walk. Every step feels like a sharp pain. (A5*,* Girl*,* 12 years)*

*When I first entered the PICU*,* I didn’t need oxygen*,* but later I started feeling really short of breath. It felt like my breath was getting shorter and shorter*,* and I couldn’t breathe properly anymore. (A3*,* Boy*,* 10 years)*

*I found that I couldn’t swallow food anymore*,* and even drinking water was difficult. Every time I tried to eat something*,* it would make me cough or feel like I was choking. (A6*,* Girl*,* 13 years)*

### Psychological distress

Children in PICUs frequently endure intense psychological distress, largely driven by physical pain and invasive medical procedures [20]. An unfamiliar hospital environment, combined with frequent examinations, often generates feelings of oppression and anxiety, fostering fear of treatment [20]. Additionally, a sense of isolation and constant monitoring can intensify emotional distress [20, 21]. A lack of emotional support and challenges in communication further amplify these feelings of helplessness, compounding the negative impact on mental health [21]. These psychological struggles often manifest as PTSD symptoms, including insomnia, mood swings, and excessive anxiety [21].

*It’s really unbearable. (A2*,* Girl*,* 9 years)*

*Every time I see that needle*,* I get a little scared. Whether it’s during treatment*,* examinations*,* or even resting*,* I always feel like I’m being watched. This makes me feel very embarrassed and uncomfortable. Each procedure causes me pain and discomfort*,* and my body hardly gets any rest. (A5*,* Girl*,* 12 years)*

*This really worried me because it’s the first time I’ve ever needed external help to breathe. (A3*,* Boy*,* 10 years)*

*This makes me feel very uncomfortable and helpless because I never imagined I would end up in this situation. (A6*,* Girl*,* 13 years)*

*This sensation makes me increasingly irritable*,* and I always feel uneasy. Even lying down feels oppressive. (A1*,* Boy*,* 8 years)*

*Whenever the doctors and nurses talk about the upcoming treatments or tests*,* I am filled with questions and worries. I don’t know what results these treatments will bring*,* and I wonder if there will be any side effects. This fear of the unknown makes me feel anxious and unable to relax. (A10*,* Boy*,* 17 years)*

*I feel trapped here*,* lonely and helpless. There’s no one to share laughter or talk with*,* and my mood keeps getting lower. (A9*,* Girl*,* 16 years)*

*Although they are helping me with my treatment*,* sometimes I feel like I’m just a machine that needs fixing. (A11*,* Boy*,* 18 years)*

### Sleep problems

Children in PICUs commonly experience sleep disturbances, often exacerbated by the hospital environment and ongoing medical procedures [22]. Factors such as alarms from monitoring devices, mechanical sounds from medical equipment, and the footsteps of healthcare staff create a noisy environment that makes it challenging for children to fall and stay asleep [22]. Additionally, intense lighting, including ceiling lights and flashing indicator lights from nearby equipment, further disrupts their ability to rest [23]. Sleep problems are closely linked to PTSD [24]. The combination of environmental stressors and frequent interruptions prevents children from achieving adequate sleep, negatively affecting both their physical recovery and mental well-being.

*In this unfamiliar place*,* I can’t sleep well. Every night*,* I hear the piercing alarms of the monitors*,* the mechanical sounds of the infusion pumps*,* and the rumbling noises of equipment being pushed by medical staff*,* accompanied by their hurried footsteps. These sounds echo around me*,* making it impossible to fall asleep. (A2*,*Girl*,* 9 years)*

*I have trouble falling asleep every night. The lights in the ward are always unbearable for me. Although the ceiling lights are dimmed*,* they remain glaring*,* with the light shining directly into my eyes. The indicator lights on the surrounding equipment keep flashing*,* with red and green lights alternating endlessly. Even when I close my eyes*,* these lights seep through my eyelids*,* making me feel uneasy and preventing me from entering deep sleep. (A1*,* Boy*,* 8 years)*

*Daytime sleep is very limited because doctors and nurses frequently come for rounds. Every time they enter*,* they turn on the room lights*,* discuss cases*,* or take notes*,* and the noisy sounds make it hard for me to sleep. When treatment begins*,* the nurses adjust my position*,* change my medication*,* or insert tubes*,* often requiring me to stay awake. Although I feel very tired*,* it is difficult for me to fall asleep in this constantly disturbed environment. (A11*,* Boy*,* 18 years)*

*I can only sleep for a few hours each night*,* and my sleep is always interrupted by the cries and noise from the other children around me. This constant noise makes it hard for me to fall asleep*,* and I often wake up*,* unable to get a full rest. (A4*,* Boy*,* 11 years)*

### Environmental stressors

In the PICU, an unfamiliar environment, ongoing medical procedures, and lack of privacy often leave children feeling vulnerable and uneasy [25]. Constant noise, harsh lighting, and elevated temperatures add to their discomfort and sense of helplessness [25]. These environmental stressors, combined with prolonged unease and the feeling of being constantly monitored, can heighten anxiety and contribute to the development of PTSD symptoms.

*I feel that the room is very stuffy*,* and the air seems stagnant. The temperature is constantly high*,* making me sweat nonstop. (A1*,* Boy*,* 8 years)* .

*There is absolutely no privacy here. The curtains in the ward provide only minimal coverage*,* and every time doctors or nurses come for rounds*,* I feel like I’m completely exposed. (A5*,* Girl*,* 12 years)*

*Every day*,* doctors and nurses perform various medical procedures. (A7*,* Boy*,* 14 years)*

*This medical environment is completely unfamiliar to me*,* and everything around me makes me feel uneasy. (A10*,* Boy*,* 17 years)*

*Every time the family members of other patients walk by*,* I feel like I’m being watched*,* and it makes me uncomfortable. I just want to hide. (A2*,*Girl*,* 9 years)*

*The light above my bed is always on. It’s a bit dim*,* but it still makes me feel uncomfortable. (A6*,* Girl*,* 13 years)*

### Medical intervention

Children in the PICU often undergo invasive medical procedures, such as venipuncture, arterial blood sampling, and nasogastric tube insertion [26]. While these interventions are critical for recovery, they frequently trigger severe psychological distress [21]. The frequency of these procedures and the constant adjustments in treatment plans leave children feeling helpless, fearful, and overwhelmed [21]. Children are especially vulnerable to such high-stress environments. Fears of needles, intubation, or suctioning magnify the sense of bodily violation, further intensifying emotional pain [27]. These experiences not only lead to the development of PTSD symptoms but also have a profound and lasting impact on their overall physical and mental health [27].

*The doctor said I need to start using oxygen to help me breathe. (A3*,* Boy*,* 10 years)* .

*The nurse started using a feeding tube to provide me with nutrition because my esophageal function has been impaired. (A6*,* Girl*,* 13 years)*

*When treatment begins*,* the nurses adjust my position*,* change my medication*,* or insert tubes*,* often requiring me to stay awake. (A11*,* Boy*,* 18 years)*

*The doctors come to check my condition and adjust the treatment plan*,* while the nurses monitor my vital signs*,* draw blood*,* and check my blood sugar. (A7*,* Boy*,* 14 years)*

*The injection fails*,* and the nurse needs to try multiple times. (A8*,* Girl*,* 15 years)*

*When suctioning*,* I feel like my throat is blocked by the tube*,* and I can hardly breathe. Every time the tube goes in*,* I can’t help but cough*,* but the more I cough*,* the worse it feels*,* and my tears just won’t stop flowing. (A5*,* Girl*,* 12 years)*

*When the nasogastric tube was inserted*,* I felt something stuck in my throat*,* making me want to vomit*,* but the nurse told me to endure it. (A1*,* Boy*,* 8 years)*

### Lack of social interaction

In the PICU, children often face a profound lack of social interaction, which deepens their emotional distress [28]. Limited family visits and minimal communication from healthcare professionals leave them feeling lonely and isolated [28]. The lively routines of school, sharing lessons, chatting with friends, and engaging in activities, are replaced by a sterile, bustling hospital environment, where medical care takes precedence over emotional connection [15]. This isolation amplifies their helplessness, fuels feelings of loneliness and anxiety, and increases the risk of developing PTSD symptoms [15].

*Although my family visits me occasionally*,* their time is always so short*,* and the rest of the time*,* I’m left alone in the hospital room. (A7*,* Boy*,* 14 years)*

*At school*,* discussing lessons*,* chatting*,* and doing activities with friends were part of my daily life*,* but now I feel isolated. I don’t have any friends around*,* and I feel especially lonely and helpless. I feel trapped in a small world*,* and there’s almost no one I can truly talk to. (A8*,* Girl*,* 15 years)*

*The doctors and nurses are always busy treating me*,* and very few take the time to sit down and talk to me or ask how I’m feeling. No one truly sits down with me*,* listens to me*,* or chats with me about light things. (A6*,* Girl*,* 13 years)*

*The other kids in the ward don’t seem to want to talk. Some of them are younger than me*,* and some are in worse condition than I am. Everyone is just lying in their beds*,* and no one talks to anyone. (A10*,* Boy*,* 17 years)*

*Mom can only visit me for a short time each day*,* and when she leaves*,* I’m left alone in bed*,* feeling like time drags on forever. Without her*,* I feel like all the treatments and pain become even harder to bear. (A5*,* Girl*,* 12 years)*

## Discussion

The findings of this study indicate that the development of PTSD symptoms among PICU patients is closely linked to their experiences during their stay, including invasive procedures, sensory overload, and emotional isolation. Children described frequent medical interventions—such as injections, suctioning, and nasogastric tube insertions—as painful and frightening, often leaving them feeling helpless and violated. Environmental stressors, including persistent noise, bright lights, and sleep disruptions, further intensified their distress. Many children expressed fear of the unknown, anxiety about treatments, and a profound sense of loneliness; some even felt they were being “repaired” rather than cared for. The lack of meaningful social interaction and emotional support exacerbated these experiences, leaving children without comfort from family or peers at a time when connection was most needed. These experiences suggest that trauma in the PICU setting arises not only from the severity of medical interventions but also from how children perceive and process their environment. The combination of physiological and emotional stress highlights the urgent need for a holistic model of care that addresses not only medical needs but also the psychological and social well-being of children [29–33].

This study further reveals that multiple stressors experienced by children in the PICU including invasive procedures, sensory overload, and emotional isolation—are significant triggers for the development of PTSD symptoms. Pediatric PTSD typically manifests in three core symptom clusters: re-experiencing, hyperarousal, and avoidance. Re-experiencing may involve intrusive memories, nightmares, or flashbacks related to painful medical procedures or frightening moments in the PICU.

Hyperarousal may present as heightened anxiety, sleep disturbances, irritability, and exaggerated startle responses, often triggered by environmental stimuli such as alarms, bright lights, or routine clinical activities. Avoidance may be reflected in the child’s reluctance to discuss their hospitalization, avoidance of medical settings, or emotional numbness.

Notably, symptom severity varied considerably among participants. While some children reported only mild symptoms such as occasional nightmares or transient fear, others described persistent and distressing symptoms across all three clusters. This variability appeared to be influenced by factors such as age, length of stay in the PICU, the intensity of medical interventions, and pre-existing vulnerabilities. Recognizing these individual differences is essential for developing personalized screening and intervention strategies, rather than adopting a one-size-fits-all approach in pediatric critical care.

Our findings deepen the understanding of how medical interventions and environmental stressors shape the psychological well-being of children in PICUs. While prior studies [1, 3, 21] have extensively documented the effects of invasive procedures and parental distress, our work highlights the often-overlooked impact of limited social interaction during hospitalization. The absence of peer or caregiver connections deprives children of critical emotional support, compounding their sense of isolation and vulnerability. Moreover, although sleep disturbances have been widely acknowledged in PICU research, their specific contribution to the development of PTSD remains underexplored. Our study highlights how disrupted sleep patterns, combined with environmental stressors such as noise, discomfort, and lack of social bonds, amplify emotional dysregulation and exacerbate psychological distress. By providing qualitative insights into the interplay among isolation, sleep disruption, and the broader PICU environment, our findings complement existing quantitative research, offering a more nuanced understanding of the multifaceted factors that contribute to the mental health challenges faced by children in critical care settings.

### Addressing researcher bias

The principal investigator’s six years of experience in the PICU, including immersive exposure through maintaining an observational presence at the bedsides of pediatric patients, provided a deep understanding of environmental stressors affecting children, such as noise, lighting, and frequent nursing procedures. This firsthand familiarity offers valuable insights into the lived experiences of patients, enabling a nuanced exploration of how these factors contribute to psychological distress. However, such proximity to the research setting also carries an inherent risk of researcher bias, as pre-existing assumptions or habitual exposure may unintentionally shape interpretations and reduce sensitivity to alternative perspectives.

To address potential biases and uphold the rigor of the study, several methodological safeguards were implemented. Triangulation was employed, integrating interviews, observations, and literature analysis to cross-validate findings and enhance reliability. Peer debriefing invited external researchers to critically review the analytical process, challenge interpretations, and ensure objectivity. Additionally, multiple researchers were involved in data analysis to validate key themes and minimize individual bias. Reflexivity further strengthened the study’s integrity, as the investigator maintained research logs to document personal assumptions and critically reflect on their influence throughout the process. These measures ensured that the investigator’s unique insights were balanced with methodological rigor, resulting in findings that were both credible and enriched by experiential depth.

### Challenges

#### Expressive limitations and supportive communication strategies

This qualitative study encountered significant challenges, particularly in capturing the voices of children with varying expressive abilities. Younger children or those experiencing emotional fluctuations often struggle to articulate their feelings and traumatic experiences, leading to the risk of incomplete or skewed data, especially when confronted with complex emotions that are difficult to verbalize. To address these limitations, we employed strategies designed to foster a supportive and accessible environment for self-expression. Simple, child-friendly language was used to explain the purpose of the interview, avoiding abstract or technical terms that might overwhelm or confuse participants. Nonverbal methods, such as illustrations and drawings, were incorporated to provide alternative avenues for children to convey their emotions visually, tapping into their creativity and comfort zones. Additionally, the researchers utilized open-ended guiding questions, such as “Do you remember what happened during your hospitalization?” to gently prompt children to share their experiences without imposing pressure. By avoiding direct inquiries about complex emotions and focusing instead on gradual, narrative-driven discussions, this study minimized anxiety while creating a safe space for children to express themselves, ultimately enriching the depth and authenticity of the collected data.

Given the wide age range of participants (8 to 18 years), developmental differences were observed and considered during data analysis. Younger children tended to express PTSD symptoms through behavioral changes and somatic complaints (e.g., stomachaches, headaches), while older adolescents were more likely to verbalize emotional distress and exhibit cognitive symptoms such as rumination and avoidance. These developmental variations underscore the importance of age-appropriate assessment and intervention strategies in clinical practice.

#### Trauma sensitivity and emotional monitoring

A key challenge in this qualitative study was managing the emotional and psychological states of the children, as revisiting traumatic experiences often triggered anxiety, distress, or even reactivation of trauma, potentially exacerbating PTSD symptoms and hindering their ability to express themselves. To address this, researchers adopted a trauma-informed approach, closely monitoring participants’ emotional cues and using strategies to mitigate distress. This included creating a safe and supportive interview environment, allowing children to take breaks as needed, and carefully phrasing questions to avoid direct or overly intrusive inquiries about trauma. In cases where signs of significant distress were observed, such as withdrawal, tearfulness, or agitation, interviews were immediately paused or terminated, and psychological support was offered to ensure the child’s well-being.

#### Methodological considerations: data saturation

Additionally, achieving data saturation posed a methodological challenge, as it required balancing thorough data collection while avoiding redundancy. To address this, researchers employed iterative data analysis throughout the study, continuously comparing new data with existing themes to identify when no new insights emerged. This process involved team discussions, peer debriefing, and cross-checking interpretations to ensure the collected data were both comprehensive and reflective of diverse experiences. These strategies reflect the careful balance between methodological rigor and ethical sensitivity necessary for research on vulnerable populations.

#### Language and communication barriers

Language and communication barriers posed a significant challenge in this study, as children’s expressive abilities varied widely across age groups, necessitating tailored approaches to facilitate meaningful dialogue. Younger children, for example, often struggle with abstract concepts or complex questions, requiring researchers to use simple, concrete, and relatable language to create a sense of comfort and trust. For an 8-year-old, questions were framed in straightforward terms, such as, “Did you sleep well in the PICU?” to encourage clear and manageable responses. In contrast, older adolescents were more capable of introspection and nuanced expression, allowing for deeper engagement through questions like, “How was your sleep during your stay in the PICU? Was it affected in any manner?” By adapting interview techniques to match the cognitive and emotional maturity of each participant, the researchers created an inclusive and supportive environment that empowered children to share their experiences authentically. This approach not only minimized communication barriers but also enriched the data by capturing perspectives across developmental stages, highlighting the importance of flexibility and sensitivity in qualitative research with diverse age groups.

#### Implications

These findings have significant implications for clinical practice in PICUs, particularly in addressing the mental health challenges faced by pediatric patients. Healthcare providers must adopt proactive strategies to mitigate environmental stressors and recognize their profound impact on psychological well-being. Specific measures, such as optimizing lighting to mimic natural circadian rhythms, minimizing noise from medical equipment, and scheduling procedures to reduce disruptions, can effectively improve sleep quality and promote emotional regulation. Additionally, encouraging family members to accompany children during hospitalization or organizing child-friendly social activities can alleviate feelings of isolation and provide emotional support. Creating a more child-centered environment is not only critical for immediate care but also essential for reducing long-term psychological distress after discharge.

Beyond environmental modifications, comprehensive psychosocial support should be the cornerstone of PICU care. In addition to medical interventions, the emotional and psychological needs of patients and their families must be integrated into holistic care plans. Nurses and other healthcare professionals can benefit from trauma-informed care training to establish stronger trust with patients. Through open communication and active listening, healthcare providers can alleviate anxiety, foster a sense of security, and provide psychological support by offering companionship, monitoring emotional changes, and explaining treatment procedures. Furthermore, the introduction of evidence-based psychosocial screening tools can help identify psychological distress in patients and their families, enabling the development of personalized intervention plans to reduce stress and enhance the care experience.

These findings also highlight the urgent need to develop evidence-based predictive models for PTSD risk in PICU patients. Such models could incorporate variables such as sleep patterns, facial expressions, PICU environmental stress, and the frequency or intensity of invasive procedures to detect early signs of psychological trauma. Early interventions can minimize psychological harm, improve recovery trajectories, and enhance the quality of life for pediatric patients after discharge. By integrating these predictive tools into clinical practice, PICUs can move toward a more preventative, patient-centered approach that prioritizes not only physical survival but also emotional recovery and psychological resilience, ensuring holistic care for critically ill children.

#### Limitations

This study employed thematic analysis to explore the experiences of children with PTSD Symptoms in the PICU and implemented several measures to ensure the reliability and validity of the findings. However, certain limitations should be acknowledged. First, the interview environment may have influenced participants’ comfort and openness in sharing their experiences. Interviews conducted in familiar and private settings were more likely to encourage candid discussions, whereas hospital-based interviews may have induced stress or recall bias, potentially limiting the depth of responses. Future studies should consider using flexible interview locations tailored to participants’ preferences to create a more supportive and relaxed atmosphere.

Second, the process of Chinese–English translation posed challenges in preserving the nuanced meaning of psychological concepts and emotional expressions. Certain terms, such as “resilience,” lack a precise equivalent in Chinese, with possible translations including “recovery ability,” “adaptability,” or “resistance to adversity,” each carrying subtly different connotations. These variations in meaning could potentially influence how participants’ responses were interpreted, as the choice of translation might emphasize certain aspects of the concept over others. To mitigate this issue, the study utilized rigorous back-translation procedures and expert reviews to enhance the accuracy and cultural appropriateness of the translated data. However, despite these measures, the potential for subtle shifts in meaning remains an inherent challenge in cross-linguistic research, which may have influenced the interpretation of nuanced psychological constructs within the data. Researchers must remain mindful of these limitations when drawing conclusions and consider the cultural context in which the data were collected.

Finally, the timing of the interviews, conducted one month after discharge, may have played a dual role in shaping the findings. On one hand, this interval allowed participants some time for emotional processing and reflection, which could have facilitated more thoughtful and comprehensive accounts of their experiences. On the other hand, it may have introduced recall bias, as participants’ recollections of traumatic experiences might have been influenced by the passage of time or their psychological recovery process. This could have affected both the accuracy and emotional intensity of their descriptions. To address these potential limitations, the study employed rigorous control measures, such as triangulation—combining data from interviews, observations, and literature to cross-verify findings—and researcher reflection to critically examine potential biases. Despite these challenges, this study provides valuable insights into the lived experiences of children with PTSD Symptoms in the PICU, offering a balanced foundation for future research and clinical intervention.

## Conclusions

This study builds on existing research by highlighting the critical, yet often overlooked, roles of social isolation and sleep disturbances in the development of PTSD among PICU patients. While prior studies have predominantly focused on medical interventions, physiological markers, and psychological factors, our findings reveal that environmental stressors, compounded by a lack of social interaction and disrupted sleep, significantly contribute to psychological distress in children. By employing rigorous qualitative methods, including triangulation and reflexivity, this study ensured the reliability and depth of its findings. These insights call for targeted interventions such as optimizing the PICU environment, incorporating comprehensive psychosocial support, and developing early predictive models to identify and mitigate PTSD risk. Future research should prioritize individualized strategies to improve the long-term psychological well-being of PICU patients, paving the way for more holistic and patient-centered care.

## Supplementary Information

Below is the link to the electronic supplementary material.

Supplementary Material 1

## Data Availability

The datasets generated and analyzed in the current study are available from the corresponding author upon reasonable request.

## Abbreviations

PICU: Pediatric intensive care units
PTSD: Post-traumatic stress disorder
COREQ: Consolidated Criteria for Reporting Qualitative Research
CRIES: Children’s Revised Impact of Event Scale

## References

[1] Ong C, Lee JH, Leow MKS, Puthucheary ZA. Functional outcomes and physical impairments in pediatric critical care survivors: A scoping review. Pediatr Crit Care Med. 2016;17:e247–59. 10.1097/PCC.0000000000000706. 27030932 10.1097/PCC.0000000000000706

[2] Davydow DS, Richardson LP, Zatzick DF, Katon WJ. Psychiatric morbidity in pediatric critical illness survivors: A comprehensive review of the literature. Arch Pediatr Adolesc Med. 2010;164:377–85. 10.1001/archpediatrics.2010.10. 20368492 10.1001/archpediatrics.2010.10 PMC2881634

[3] Als LC, Picouto MD, Hau MD, Nadel SM, Cooper S, Pierce M. Mental and physical well-being following admission to pediatric intensive care. Pediatr Crit Care Med. 2015;16:e141–9. 10.1097/PCC.0000000000000424. 25901544 10.1097/PCC.0000000000000424

[4] Corbet Burcher G, Picouto MD, Als LC, Cooper M, Pierce CM, Nadel S, et al. Post-traumatic stress after PICU and corticosteroid use. Arch Dis Child. 2018;103:887–9. 10.1136/archdischild-2017-314157. 29175821 10.1136/archdischild-2017-314157

[5] Kim SJ, Yeo JH. Factors affecting posttraumatic stress disorder in South Korean trauma nurses. J Trauma Nurs. 2020;27:50–7. 10.1097/JTN.0000000000000482. 31895320 10.1097/JTN.0000000000000482

[6] Duffy E, Avalos G, Dowling M. Secondary traumatic stress among emergency nurses: a cross-sectional study. Int Emerg Nurs. 2015;23:53–8. 10.1016/j.ienj.2014.05.001. 24927978 10.1016/j.ienj.2014.05.001

[7] Hamblen J, Barnett E. PTSD in children and adolescents. N Atl Cent PTSD. 2025. website.

[8] Merikangas KR, He JP, Burstein M, Swanson SA, Avenevoli S, Cui L, et al. Lifetime prevalence of mental disorders in U.S. adolescents: results from the National comorbidity Survey Replication—adolescent Supplement (NCS-A). J Am Acad Child Adolesc Psychiatry. 2010;49:980–9. 10.1016/j.jaac.2010.05.017. 20855043 10.1016/j.jaac.2010.05.017 PMC2946114

[9] Frem T. PTSD in preschool children: a review and synthesis of research on the need for a separate DSM category, 36;2013. https://concept.journals.villanova.edu/index.php/concept/article/view/1529/1346.

[10] De Young AC, Landolt MA. PTSD in children below the age of 6 years. Curr Psychiatry Rep. 2018;20:97. 10.1007/s11920-018-0966-z. 30221307 10.1007/s11920-018-0966-z

[11] Doyle L, McCabe C, Keogh B, Brady A, McCann M. An overview of the qualitative descriptive design within nursing research. J Res Nurs. 2020;25:443–55. 10.1177/1744987119880234. 34394658 10.1177/1744987119880234 PMC7932381

[12] Tong A, Sainsbury P, Craig J. Consolidated criteria for reporting qualitative research (COREQ): a 32-item checklist for interviews and focus groups. Int J Qual Health Care. 2007;19:349–57. 10.1093/intqhc/mzm042. 17872937 10.1093/intqhc/mzm042

[13] Francis JJ, Johnston M, Robertson C, Glidewell L, Entwistle V, Eccles MP, et al. What is an adequate sample size? Operationalising data saturation for theory-based interview studies. Psychol Health. 2010;25:1229–45. 10.1080/08870440903194015. 20204937 10.1080/08870440903194015

[14] de Pellegars A, Cariou C, Le Floch M, Duverger P, Boussicault G, Riquin E. Risk factors of post-traumatic stress disorder after hospitalization in a pediatric intensive care unit: a systematic literature review. Eur Child Adolesc Psychiatry. 2024;33:2991–3001. 10.1007/s00787-023-02141-8. 36739584 10.1007/s00787-023-02141-8

[15] Tang MT, Chui PL, Chong MC, Zhang HY, Li XM, Wang T. Translation, cross-cultural adaptation, reliability, and validity of the Chinese version of the intensive care unit environment stress scale for pediatric patients. J Pediatr Nurs. 2024;77:e511–9. Epub. PMID: 38782669. 38782669 10.1016/j.pedn.2024.05.017

[16] Creswell JW. Research Design: Qualitative, Quantitative and Mixed Methods Approaches. Los Angeles: Sage; 2009.

[17] Houghton C, Casey D, Shaw D, Murphy K. Rigour in qualitative case-study research. Nurse Res. 2013;20:12–7. 10.7748/nr2013.03.20.4.12.e326. 23520707 10.7748/nr2013.03.20.4.12.e326

[18] Vaismoradi M, Turunen H, Bondas T. Content analysis and thematic analysis: implications for conducting a qualitative descriptive study. Nurs Health Sci. 2013;15:398–405. 10.1111/nhs.12048. 23480423 10.1111/nhs.12048

[19] Quadir A, Festa M, Gilchrist M, Thompson K, Pride N, Basu S. Long-term follow-up in pediatric intensive care-a narrative review. Front Pediatr. 2024;12:1430581. 10.3389/fped.2024.1430581. PMCID: PMC11246917. 10.3389/fped.2024.1430581 PMC11246917 39011062

[20] Ko MSM, Poh PF, Heng KYC, Sultana R, Murphy B, Ng RWL, et al. Assessment of long-term psychological outcomes after pediatric intensive care unit admission: a systematic review and meta-analysis. JAMA Pediatr. 2022;176:e215767. 10.1001/jamapediatrics.2021.5767. 35040918 10.1001/jamapediatrics.2021.5767 PMC8767488

[21] Tang M, Chui PL, Chong MC, Liu X. Post-traumatic stress disorder in children after discharge from the pediatric intensive care unit: a scoping review. Eur Child Adolesc Psychiatry. 2025;34:483–96. 10.1007/s00787-024-02505-8. ePub 2024 Jun 25. PMID: 38916767. 38916767 10.1007/s00787-024-02505-8

[22] Kalvas LB, Harrison TM, Solove S, Happ MB. Sleep disruption and delirium in critically ill children: study protocol feasibility. Res Nurs Health. 2022;45:604–15. 10.1002/nur.22259. Epub. PMCID: PMC9529999. 10.1002/nur.22259 PMC9529999 35986659

[23] Siraji MA, Spitschan M, Kalavally V, Haque S. Light exposure behaviors predict mood, memory and sleep quality. Sci Rep. 2023;13:12425. 10.1038/s41598-023-39636-y. 37528146 10.1038/s41598-023-39636-y PMC10394000

[24] Koffel E, Khawaja IS, Germain A. Sleep disturbances in posttraumatic stress disorder: updated review and implications for treatment. Psychiatr Ann. 2016;46:173–6. 10.3928/00485713-20160125-01. Epub. PMCID: PMC5068571. 27773950 10.3928/00485713-20160125-01 PMC5068571

[25] Cardoso SB, Oliveira ICDS, Souza TV, Carmo SAD. Pediatric intensive care Unit: reflection in the light of Florence Nightingale’s environmental theory. Rev Bras Enferm. 2021;74:e20201267. English, Portuguese. 10.1590/0034-7167-2020-1267. PMID: 34320154. 10.1590/0034-7167-2020-1267 34320154

[26] Seyedhejazi M, Hamidi M, Sheikhzadeh D, Aliakbari Sharabiani B. Nasogastric tube placement errors and complications in pediatric intensive care unit: a case report. J Cardiovasc Thorac Res. 2011;3:133–4. Epub. PMCID: PMC3825344. 24250971 10.5681/jcvtr.2011.029 PMC3825344

[27] Birnie KA, Noel M, Chambers CT, Uman LS, Parker JA. Psychological interventions for needle-related procedural pain and distress in children and adolescents. Cochrane Database Syst Rev. 2018;10:CD005179. 10.1002/14651858.CD005179.pub4. PMCID: PMC6517234. 10.1002/14651858.CD005179.pub4 PMC6517234 30284240

[28] Costello JM, Patak L, Pritchard J. Communication vulnerable patients in the pediatric ICU: enhancing care through augmentative and alternative communication. J Pediatr Rehabil Med. 2010;3:289–301. 10.3233/PRM-2010-0140. PMID: 21791863. 10.3233/PRM-2010-0140 21791863

[29] Colville G, Kerry S, Pierce C. Children’s factual and delusional memories of intensive care. Am J Respir Crit Care Med. 2008;177:976–82. 10.1164/rccm.200706-857OC. 18244955 10.1164/rccm.200706-857OC

[30] Dow BL, Kenardy JA, Le Brocque RM, Long DA. Cognitive predictors of posttraumatic stress in children 6 months after Paediatric Intensive Care Unit admission. Trauma Care. 2023;3:82–92. 10.3390/traumacare3020009.

[31] Long D, Gibbons K, Le Brocque R, Schults JA, Kenardy J, Dow B. Midazolam exposure in the paediatric intensive care unit predicts acute post-traumatic stress symptoms in children. Aust Crit Care. 2022;35:408–14. 10.1016/j.aucc.2021.06.004. 34373171 10.1016/j.aucc.2021.06.004

[32] Rennick JE, Morin I, Kim D, Johnston CC, Dougherty G, Platt R. Identifying children at high risk for psychological sequelae after pediatric intensive care unit hospitalization. Pediatr Crit Care Med. 2004;5:358–63. 10.1097/01.pcc.0000128603.20501.0d. 15215006 10.1097/01.pcc.0000128603.20501.0d

[33] Als LC, Picouto MD, O’Donnell KJ, Nadel S, Cooper M, Pierce CM, et al. Stress hormones and posttraumatic stress symptoms following paediatric critical illness: an exploratory study. Eur Child Adolesc Psychiatry. 2017;26:511–9. 10.1007/s00787-016-0933-3. 27995329 10.1007/s00787-016-0933-3 PMC5394132

