# Supplementary material for: Post-traumatic stress disorder symptoms in children after PICU discharge: exploring contributing factors and the need for targeted interventions
Source: BMC Psychiatry. 2026 Apr 24;26:451. doi: 10.1186/s12888-026-08079-w (PMC13244976; doi:10.1186/s12888-026-08079-w)
Supplement: Supplementary file 1 — Supplementary Material 1 [file 12888_2026_8079_MOESM1_ESM.docx]

Table A. Interview questions.

| Warm-up | | |
| --- | --- | --- |
| Topic | Children’s group (8-12 years) | Adolescents’ group (13-18 years) |
| Self-introduction | "Hello, I’m Tang MaoTing, a nurse researching this topic. It’s nice to meet you!" | "Hi, I’m Tang MaoTing. Today, we’d like to talk about your experience in the PICU. Your sharing may help more people." |
| Building trust | "What fun thing did you do before coming here today?" | "How have things been at school/home recently?" |
| Interview principles | "If there are any questions that make you feel uncomfortable, you can tell me anytime." | "If there are any questions you don’t want to answer, let me know, and we’ll skip them." |
| Core questions | | |
| Topic | Children’s group (8-12 years) | Adolescents’ group (13-18 years) |
| PICU hospitalization experience | "Do you remember what happened during your hospitalization?" | "Can you recall your experience in the PICU? You can start from the moment you were admitted." |
|  | "What do you think about the PICU? Was it what you expected?" | "How did the PICU environment make you feel?" |
| Physical and emotional feelings | "Did you feel any discomfort while in the PICU?" | "How did you feel physically and emotionally during your stay in the PICU?" |
|  | "Were you scared at that time? What made you feel afraid?" | "Did you ever feel anxious, scared, or distressed?" |
| Impact of the PICU environment | "Were there any sounds or lights in the PICU that made you feel scared?" | "Was there anything in the PICU environment that made you feel uncomfortable or stressed?" |
|  | "Was there anything that made you feel a little happier?" | "Was there anyone or anything that made you feel a bit better while in the PICU?" |
| Sleep conditions | "Did you sleep well in the PICU?" | "How was your sleep during your stay in the PICU? Was it affected in any way?" |
|  | "Was there anything that kept you from sleeping?" | "Has your sleep pattern changed because of your PICU experience?" |
| Fears and worries | "What was the scariest thing for you in the PICU?" | "During your stay in the PICU, was there anything you were particularly worried or afraid about?" |
|  | "What did you do when you felt scared?" | "How did you cope with your fear at that time?" |
| Current emotions and adaptation | "Do you feel different now compared to before your PICU stay?" | "Has your experience in the PICU affected your emotions and life now?" |
|  | "Do you still often think about what happened in the PICU?" | "Have these experiences impacted your daily life, such as studying or socializing?" |
| Probing questions | | |
| Topic | Children’s group (8-12 years) | Adolescents’ group (13-18 years) |
| Impact of medical staff | "Was there a doctor or nurse in the PICU who made you feel especially good?" | "What impression do you have of the medical staff in the PICU?" |
| Social support | "Did you talk to your parents or friends about your time in the PICU?" | "Do you talk to your friends or family about your PICU experience?" |
| Future advice | "If another child had to go to the PICU, what would you like to tell them?" | "If your friend had to go to the PICU, what advice would you give them?" |
| Closing | | |
| Topic | Children’s group (8-12 years) | Adolescents’ group (13-18 years) |
| Expressing gratitude | "You did a great job sharing today! Thank you!" | "Thank you for sharing, this is really important for our research." |
| Checking emotional state | "Did these questions make you feel upset?" | "Did any of these questions make you feel uncomfortable? If so, we can talk more about it." |
| Ending on a positive note | "What fun things are you going to do next?" | "Have you been into any hobbies or activities lately?" |
